# A national survey of hospital readiness during the COVID-19 pandemic in Nigeria

**DOI:** 10.1371/journal.pone.0257567

**Published:** 2021-09-21

**Authors:** Dimie Ogoina, Dalhat Mahmood, Abisoye Sunday Oyeyemi, Ogochukwu Chinedum Okoye, Vivian Kwaghe, Zayaid Habib, Uche Unigwe, Michael Onyebuchi Iroezindu, Musa Abubakar Garbati, Stella Rotifa, Olukemi Adekanmbi, Iliyasu Garba, Farouq Muhammad Dayyab, Sanusi Mohammed Ibrahim, Ibrahim Musa Kida, Adamu Adamu, Datonye Alasia, Sati Klein Awang, John Oghenevwirhe Ohaju-Obodo, Rabi Usman, Yahaya Mohammed, Ayanfe Omololu, Ekaete Alice Tobin, Sylvanus Okogbenin, Danny Asogun, Iraoyah Kelly, Bala Waziri, Aliyu Mamman Nauzo, Yusuf Jibrin, Abdulrazaq Garba Habib

**Affiliations:** 1 Department of Internal Medicine, Niger Delta University/Niger Delta University Teaching Hospital, Yenagoa, Bayelsa State, Nigeria; 2 African Field Epidemiology Network, Abuja, Nigeria; 3 Department of Community Medicine, Niger Delta University/Niger Delta University Teaching Hospital, Yenagoa, Bayelsa State, Nigeria; 4 Department of Internal Medicine, Faculty of Clinical Medicine, Delta State University, Abraka, Delta State, Nigeria; 5 Department of Internal Medicine, University of Abuja Teaching Hospital, Federal Capital Territory, Gwagwalada, Nigeria; 6 Department of Medicine, University of Nigeria Teaching Hospital, Ituku/Ozalla, Enugu, Nigeria; 7 University of Maiduguri/University of Maiduguri Teaching Hospital, Maiduguri, Borno State, Nigeria; 8 Department of Community Medicine, Federal Medical Centre Yenagoa, Yenagoa, Bayelsa State, Nigeria; 9 Department of Medicine, University of Ibadan/University College Hospital, Ibadan, Oyo State, Nigeria; 10 Infectious Disease and Tropical Medicine Unit, Department of Medicine, College of Health Sciences, Bayero University Kano, Kano, Nigeria; 11 Infectious Diseases Hospital, Kano, Nigeria; 12 Department of Internal Medicine, College of Health Sciences University of Port Harcourt, Port Harcourt, Nigeria; 13 Department of Internal Medicine, Federal Medical Centre, Yola, Adamawa State, Nigeria; 14 Zamfara State Ministry of Health, Zamfara, Nigeria; 15 Department of Medical Microbiology and Parasitology, Usmanu Danfodiyo University Sokoto, Sokoto, Nigeria; 16 Department of Medicine, Federal Medical Centre Abeokuta, Ogun State, Nigeria; 17 Institute of Lassa fever research and control, Irrua Specialist Teaching Hospital, Irrua, Nigeria; 18 Department of Obstetrics and Gynaecology, Irrua Specialist Teaching Hospital, Irrua, Nigeria; 19 Dept of Community Health, Irrua Specialist Teaching Hospital, Irrua, Nigeria; 20 Department of Internal Medicine, Irrua Specialist Teaching Hospital, Irrua, Nigeria; 21 Department of Medicine, Ibrahim Badamasi Babangida Specialist Hospital, Minna, Niger State, Nigeria; 22 Department of Paediatrics, Federal Medical Centre Birnin Kebbi, Kebbi State, Nigeria; 23 Department of Medicine, Abubakar Tafawa Balewa University Teaching Hospital Bauchi, Bauchi, Nigeria; University of South Carolina College of Pharmacy, UNITED STATES

## Abstract

**Introduction:**

The COVID-19 pandemic continues to overwhelm health systems across the globe. We aimed to assess the readiness of hospitals in Nigeria to respond to the COVID-19 outbreak.

**Method:**

Between April and October 2020, hospital representatives completed a modified World Health Organisation (WHO) COVID-19 hospital readiness checklist consisting of 13 components and 124 indicators. Readiness scores were classified as adequate (score ≥80%), moderate (score 50–79.9%) and not ready (score <50%).

**Results:**

Among 20 (17 tertiary and three secondary) hospitals from all six geopolitical zones of Nigeria, readiness score ranged from 28.2% to 88.7% (median 68.4%), and only three (15%) hospitals had adequate readiness. There was a median of 15 isolation beds, four ICU beds and four ventilators per hospital, but over 45% of hospitals established isolation facilities and procured ventilators after the onset of COVID-19. Of the 13 readiness components, the lowest readiness scores were reported for surge capacity (61.1%), human resources (59.1%), staff welfare (50%) and availability of critical items (47.7%).

**Conclusion:**

Most hospitals in Nigeria were not adequately prepared to respond to the COVID-19 outbreak. Current efforts to strengthen hospital preparedness should prioritize challenges related to surge capacity, critical care for COVID-19 patients, and staff welfare and protection.

## Introduction

The coronavirus disease 2019 (COVID-19) pandemic is currently ravaging countries across the world, including Nigeria [1]. The World Health Organisation (WHO) had earlier categorised Nigeria as a high-risk country for the spread of the disease in view of its weak healthcare system, large population, and sizeable travelling population across various international destinations [2, 3].

The first case of COVID-19 in Nigeria was reported on the 27^th^ of February 2020 [4]. As of 25^th^ April 2021, Nigeria has reported 164,684 confirmed cases and 2,061 deaths due to COVID-19 [5]. During the first few months of the COVID-19 pandemic in Nigeria, there were disruptions in health service delivery across the country, limited availability of isolation facilities and apparent inadequate preparedness of hospitals to respond to the outbreak. The public health response to the COVID-19 in Nigeria [6], and some aspects of public health preparedness towards COVID-19 outbreak in Nigeria have been previously described [7, 8]. However, none of these studies directly accessed the readiness of hospitals to respond the COVID-19 pandemic on a national scale. Inadequate readiness of hospitals to respond to the COVID-19 pandemic may lead to inadvertent exposure of healthcare workers, poor outcomes among hospitalised patients and disrupt routine delivery of essential health services [3, 9, 10].

In this study, we determined the level of readiness of hospitals in Nigeria to respond to the COVID-19 pandemic. The outcome of this assessment will provide knowledge to guide interventions to improve preparedness for the sustained pandemic response and determine areas of priority for intervention in the country.

## Methods

### Study design

A descriptive cross-sectional study was conducted among public healthcare facilities in Nigeria. Beginning in April 2020, we invited secondary and tertiary healthcare facilities designated as COVID-19 treatment centres across all six geopolitical zones of the country to participate in the study. At the time of the study, the 36 states and the Federal Capital Territory (FCT) of Nigeria had a total of 68 designated public funded hospital-based COVID-19 treatment centre located within secondary or tertiary health facilities, with at least one treatment centres per state. We invited all 68 public hospitals to participate in this study by contacting the head of treatment centre in each hospital. Participating hospitals were selected by purposive sampling, based on their willingness to participate.

### Data collection

A total of 20 of the 68 treatment centres agreed to participate in the study. Information on hospital characteristics, such as availability of beds, isolation units, intensive care units (ICU), ventilators, and specialist COVID-19-related health workforce, were obtained using a structured questionnaire. Data on hospital readiness was obtained using a modified WHO COVID-19 hospital readiness checklist (interim version February 2020). The WHO readiness checklist was developed to guide hospitals to define and initiate priority actions needed to ensure a rapid response to the COVID-19 outbreak. This original checklist consisted of 11 key components; under each component, there is a list of questions (or indicators) regarding the status of implementation of the recommended action specific to that component. We modified the checklist by adding two more components: staff welfare and availability of critical items (S1 Text). These are also essential aspects of hospital preparedness as identified by the WHO [5, 7–9]. The indicators for the 11 components were rephrased for clarity and questions for the last two components were developed after reviewing published recommendations from the WHO [5, 7–9]. Overall, the modified checklist had 13 components and 124 indicators. The study checklist and questionnaire was pre-tested in one of the COVID-19 treatment center and this health facility was excluded from participating in the survey.

Hard copies of the questionnaire and checklist were printed out from soft copies send to each participating hospital and completed by one or more representatives of the hospital (i.e., Medical Director, or Head of Clinical Services or Head of COVID-19 response team, etc.) who had necessary information about service delivery in the hospital and preparations toward the COVID-19 pandemic.

### Scoring

Responses to each indicator on the checklist were classified into four groups: not started, due for review, in progress and completed (NB. the responses in the original checklist did not include “not started”). A response of “completed’ was scored 2, “in progress” and “due for review” were scored 1 each, and “not started” was scored 0. The scores for each component and the overall score were used to calculate percentage readiness scores.

The readiness scores were classified as adequate (≥80%), moderate (50–79%) and limited/not ready (<50%), using previously established WHO scoring classification [10].

### Data analysis

Data from each centre were entered, cleaned, aggregated, and analysed using Microsoft Excel software. Research teams in each hospital ensured that all responses in the checklist and questionnaire were answered before data entry to avoid missing data. Descriptive statistics were computed for continuous variables and presented using median (range) while frequencies and proportions were generated for categorical variables. Results were presented using tables and chart.

### Ethics approval and consent to participate

Ethical approval for the study was obtained from the National Health Research and Ethics Committee. (NHREC/01/01/2007-14/04/2020). Individual informed consent was not required because only one individual completed the questionnaire for each institution (hospital) and the hospital had given consent for the questionnaire to be completed. The Informed Consent procedure for our research was approved by National Health Research and Ethics Committee Nigeria (NHREC/01/01/2007-14/04/2020).

## Results

### Characteristics of participating hospitals

The characteristics of participating hospitals are summarised in Table 1. A total of 20 hospitals across all six geopolitical zones of the country, comprising 17 tertiary health facilities and three secondary health facilities, participated in the study. All hospitals were designated as isolation/COVID-19 treatment centres by Federal and State health authorities. Of the 20 hospitals 13 (65%) were Federal hospitals while seven (35%) hospitals were managed by State governments. The hospitals had a total bed capacity of 8746 beds, with a median (range) of 388 beds (150–800 beds). As of the time of evaluation, 18 hospitals had admitted a suspected or confirmed case of COVID-19.

**Table 1:** COVID-19 hospital readiness in Nigeria: Characteristics of hospital surveyed.

Characteristics	N = 20 hospitals n (%)	Median	Range	Total
**Administrative status**				
Federal	13 (65)			
State	7 (35)			
**Level of care**				
Secondary	3 (15)			
Tertiary	17 (85)			
**Features in respect of care of COVID 19**				
Hospital has admitted suspected or confirmed cases	18 (90)			
Hospital with functional IPC Committee	14 (70)			
Hospitals established Isolation unit on account of COVID-19	9 (45)			
Beds in hospital	20 (100.0)	388	150–800	8746
Beds in isolation units	18 (90)	15.0	2–94	381
Beds in Adult Isolation units	18 (90)	13.0	2–94	360
Beds in Paediatric Isolation units	4 (20)	3.0	2–9	21
Beds in a single room	12 (60)	2.0	1–8	34
Beds in ICU	16 (80)	4	3–12	95
Beds in Adult ICU	16 (80)	5	3–8	74
Beds in paediatric ICU	4 (20)	3	1–4	11
Functional Ventilators	17 (85)	4	1–14	96
Ventilators procured due to COVID-19	13(65)	1	1–11	54
**Human resource for COVID-19 response**				
**Anaesthesiologists**				
Full term	17 (85)	4	1–14	80
Trained for COVID 19 intervention	8 (40)	2	1–6	23
**Infectious disease physicians**				
Full term	11 (55)	1	1–5	20
Trained for COVID 19 intervention	10 (50)	1	1–4	18
**Public health physicians**				
Full term	16 (80)	7	1–28	111
Trained for COVID 19 intervention	10 (50)	2	1–7	39
**Pulmonologists**				
Full term	15 (75)	1	1–3	20
Trained for COVID 19 intervention	10 (50)	1	1–2	14
**Clinical microbiologists**				
Full term	13 (65)	1	1–7	37
Trained for COVID 19 intervention	9 (45)	1	1–5	16
**Infection control officer**				
Full term	16 (80)	2	1–6	47
Trained for COVID 19 intervention	13 (65)	2	1–5	38

Key: N-number of study participants, ICU-intensive care unit, IPC-infection prevention and control

### COVID-19-related infrastructure and equipment

Most of the hospitals (18 of 20) had designated isolation units, but nine (45%) of these of hospitals established isolation units after the onset of the COVID-19 outbreak in Nigeria. Sixteen hospitals had functional (ICU), with a median of four ICU beds per hospital. Seventeen hospitals had functional ventilators for all types of patient care, with a total of 96 ventilators, and a median of four ventilators per hospital. Fifty-four (56.3%) of the ventilators were procured for COVID-19 response.

### COVID-19-related specialist human resources

The total number of full-time specialist human resource for COVID-19 response ranged from 20 for infectious diseases (ID) physicians to 111 for public health physicians (Table 1). Majority of hospitals (17 of 20) had at least one anaesthesiologist on full term employment, while only 11 hospitals had ID physicians on full term employment. About 90% of ID physicians had been trained on COVID-19 intervention, while about 53.2% of public health physicians and 28.8% of anaesthesiologists had prior training for COVID-19 intervention.

### Hospital readiness scores

The compliance of the 20 hospitals with the modified WHO hospital readiness checklist for COVID-19 response is summarised in Table 2. The readiness scores ranged from 28.2% to 88.7%. Three (15%) hospitals had ‘adequate’ readiness, 15 (75%) had ‘moderate’ readiness and two (10%) hospitals were classified as ‘not ready’. Of the three secondary health facilities studied, one had ‘adequate’ readiness score, one was ‘moderately ready’ and the third was ‘not ready’. Of the three hospitals with ‘adequate’ readiness, two were evaluated before June 2020, while one was evaluated after June 2020.

**Table 2:** Compliance with 13 components of modified WHO COVID-19 readiness checklist among 20 hospitals in Nigeria.

		Readiness components													
		IMT	SC	IPC	CM	HR	CEHR	SUR	CMM	LSP	LS	ESS	SW	CI	Overall readiness
Name of Hospital	Date of evaluation	%	%	%	%	%	%	%	%	%	%	%	%	%	%
UDUTH	01.04.20	50	100	70	80.8	81.8	75.0	88.9	100	75.0	100	71.4	40	77.3	80.2
DELSUTH	14.04.20	50	77.8	100	100	68.2	87.5	83.3	80	62.5	60	57.1	100	36.4	76.2
FMCA	20.04.20	16.7	11.1	32.5	11.5	0	25	38.9	45	58.3	45	21.4	0	63.6	31.1
AKTH	28.04.20	66.7	83.3	85	69.2	40.9	50	55.6	70	66.7	35	42.9	0	50	59.7
CHW	06.05.20	66.7	55.6	92.5	88.5	68.2	87.5	83.3	95	79.2	85	71.4	100	68.2	81.1
ATBUTH	15.05.20	50	83.3	85.0	80.8	59.1	62.5	61.1	50	66.7	65	71.4	30	50	66.5
NDUTH	16.05.20	100	66.7	95.0	69.2	59.1	100	77.8	70	62.5	90	28.6	50	31.8	69.4
UATH	25.05.20	33.3	66.7	97.5	76.9	81.8	100	83.3	80	70.8	85	100	70	22.7	76.6
FMCBK	25.05.20	66.7	88.9	85	76.9	40.9	100	77.8	55	58.3	70	78.6	30	36.4	66.9
UPTH	26.05.20	100	55.6	85	80.8	86.4	62.5	77.8	100	87.5	100	78.6	60	45.5	79.4
GHG	16.06.20	16.7	27.8	40	15.4	18.2	25.0	5.6	20	41.7	5.0	7.1	0	95.5	28.2
ASYBSHG	17.06.20	100	55.6	85	84.6	77.3	100	94.4	85	83.3	65	100	60	45.5	78.2
UCH	01.08.20	50	61.1	62.5	76.9	50	50	100	75	70.8	95	71.4	40	31.8	66.1
UMTH	17.08.20	100	77.8	92.5	96.2	90.9	87.5	100	90	100	100	78.6	80	54.6	88.7
UNTH	28.08.20	66.7	88.9	92.5	73.1	59.1	87.5	100	65.0	45.8	80	71.4	80	45.5	73.4
ISTH	10.09.20	100	61.1	87.5	76.9	77.3	62.5	83.3	85.0	75	90	92.9	30	59.1	77
AIFUTH	18.09.20	100	44.4	75	73.1	40.9	75.0	61.1	55.0	58.3	75	64.3	50	68.2	63.7
ESUTH	08.10.20	50	50	50	50	40.9	50	72.2	80	54.2	40	71.4	60	27.3	52.4
FMCY	13.10.20	50	55.6	100	73.1	59.1	87.5	100	65	41.7	40	28.6	50	77.3	67.3
GHM	29.10.20	66.7	55.6	67.5	61.5	59.1	25	72.2	55	45.8	65	42.9	20	31.8	54.4
All Hospitals		66.7	61.1	85	76.9	59.1	75	80.6	72.5	64.6	72.5	71.4	50	47.7	68.4

NB: **Names of hospitals**: Usman Danfodio University Teaching Hospital (UDTH), Sokoto, Delta State University Teaching Hospital, Delta (DELSUTH), Federal Medical Centre Abeokuta, Ogun State (FMCA), Aminu Kano Teaching Hospital, Kano (AKTH), Niger Delta University Teaching Hospital, Bayelsa (NDUTH), Central Hospital, Warri, Delta (CHW), Abubakar Tafewa Balewa University Teaching Hospital, Bauchi (ATBUTH), University of Port Harcourt Teaching Hospital, Rivers (UPTH), University of Abuja Teaching Hospital, FCT (UATH), Federal Medical Centre Birnin Kebbi (FMCBK), General Hospital Gusau, Zamfara (GHG), Ahmed Sani Yariman Bakura Specialist Hospital, Zamfara (ASYBSH), University College Hospital Ibadan, Oyo (UCH), University of Maiduguri Teaching Hospital, Borno (UMTH), University of Nigeria Teaching Hospital, Enugu (UNTH), Alex Ekwueme Fed. University Teaching Hospital, Eboinyi (AEFUTH), Irrua Specialist Teaching Hospital, Edo (ISTH), Enugu State University Teaching Hospital, Enugu (ESUTH), Federal Medical Centre Yenagoa, Bayelsa (FMCY), General Hospital, Minna, Niger State.

**Components of readiness checklist**: IMT (Incident Management Team), SC (Surge Capacity), IPC (Infection Prevention and Control), CM (Case Management), HR (Human Resources), CEHR (Continuity of Essential Health Services and Patient Care), SUR (Surveillance: Early Warning Sign and Monitoring), CMM (Communication), LSP (Logistic and Management of Supplies, Including Pharmaceuticals), LS (Laboratory Services), ESS (Essential Support Services), SW (Staff Welfare), CI (Critical Items).

### Components of hospital readiness for COVID 19

**Fig 1:**
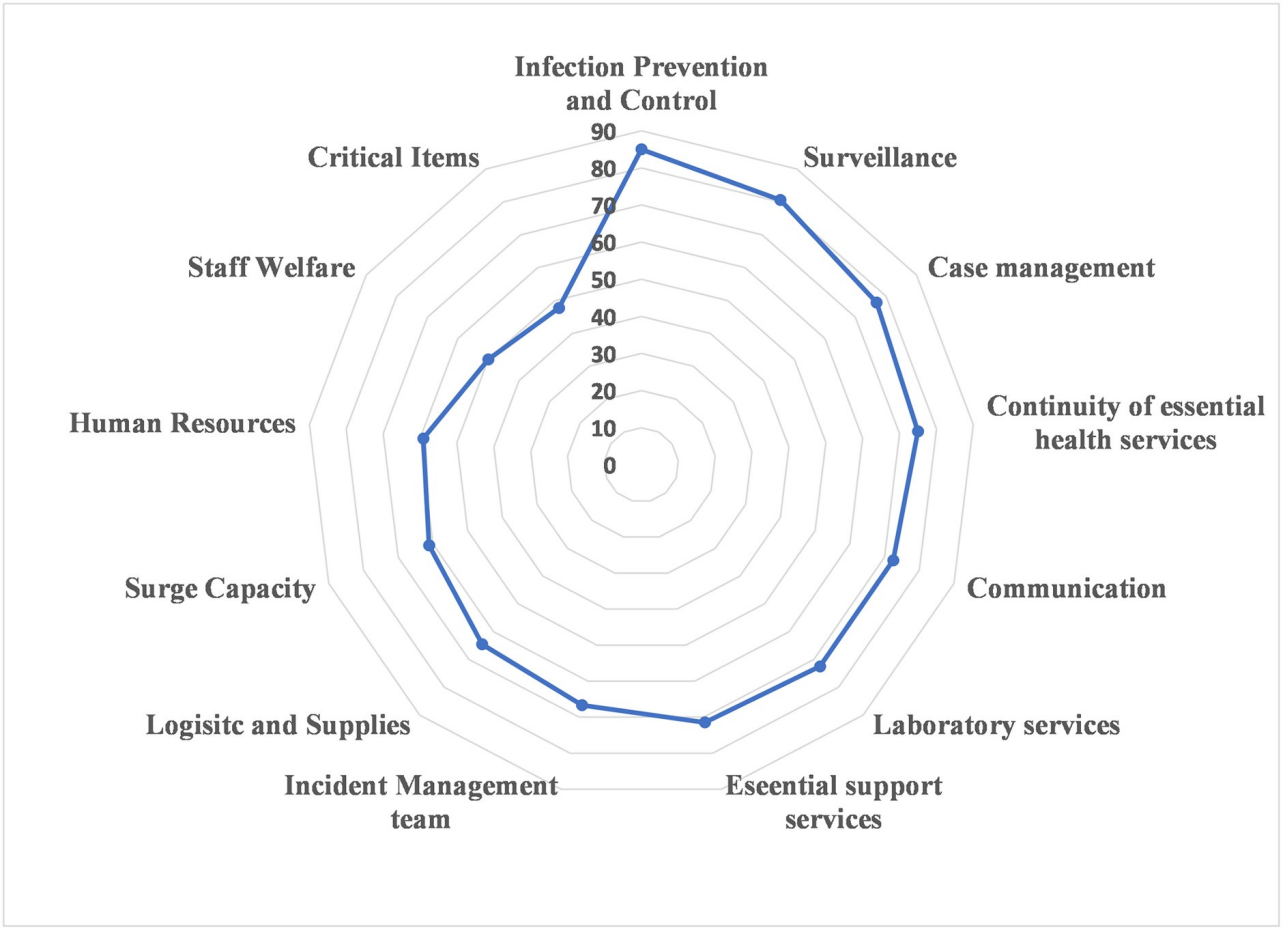
Radar chart of readiness scores (%) of 13 components of WHO modified COVID-19 hospital readiness checklist among 20 hospitals in Nigeria.

The overall readiness scores according to the various components of the readiness checklist ranged from 47.7% to 85% (Fig 1). Infection prevention and control (85%) and surveillance (80.6%) had the highest readiness scores, while staff welfare (50%) and availability of critical items (47.7%) had the lowest readiness scores. The following section outlines the level of implementation of indicators in each component of the readiness checklist.

### Incident management team and surge capacity

Only 14 (70%) of the 20 hospitals evaluated had established an incident management committee, and 40% and 35% had an emergency response plan and an emergency operation centre, respectively (Table 3).

**Table 3:** Implementation of incident management plan and surge capacity readiness indicators among 20 hospitals in Nigeria.

Sn	Hospital readiness indicator	Completed	In progress	Due for review	Not started
		%	%	%	%
	**Incident management plan**				
1	Hospital has an Emergency Response Plan	40	40	5	15
2	Hospital has an Emergency operation centre	35	25	5	35
3	Hospital has established an incident management committee or response team for COVID-19	70	20	5	5
	**Surge capacity**				
1	Hospital has estimated its capacity to accommodate COVID-19 surge	45	5	40	10
2	Hospital has identified ways of expanding hospital in-patient capacity	50	0	40	10
3	Hospital has identified potential gaps in the provision of health care, especially critical care	40	5	50	5
4	Hospital has developed strategies to address challenges associated with critical care	35	5	55	5
5	Hospital has considered outsourcing care of non-critical patients to alternative treatment sites	25	0	35	40
6	Hospital, in conjunction with local authorities, has identified other sites for patient care	20	10	15	55
7	Hospital has considered cancelling nonessential services (e.g., elective surgery) when necessary	80	5	10	5
8	Hospital has prioritized patient care according to available treatment capacity and demand	75	0	20	5
9	Hospital has considered setting up isolation tents to cater for a surge in patients’ numbers	30	10	35	25

Regarding surge capacity, most hospitals (80%) had cancelled nonessential services, but less than half of the hospitals have estimated their capacity to accommodate COVID-19 cases and identified ways of expanding hospital in-patient capacity. Only seven (35%) hospitals had developed strategies to address challenges associated with critical care (Table 3).

### Infection prevention and control (IPC)

Most hospitals had completed indicators related to IPC, including training of healthcare workers (HCW) on IPC (75%), provision of IPC resources (80%) and compliance with isolation (80%), and droplet/contact precautions (80%) by HCW (Table 4). Most hospitals (80%) also have a team of HCW designated to care exclusively for suspected and confirmed cases of COVID 19. However, only 50% of hospitals had certified that HCW are applying standard precautions, while only 40% reported having adequate personal protective equipment (PPE) for HCW.

**Table 4:** Implementation of infection prevention and control readiness indicators among 20 hospitals in Nigeria.

Sn	Hospital readiness indicator	Completed	In progress	Due for review	Not started
		%	%	%	%
1	Hospital sensitized HCWs, patients, and visitors about respiratory and hand hygiene and prevention of HCAI	80	0	20	0
2	Hospital has provided soap and water for hand hygiene	80	0	20	0
3	Hospital has provided alcohol-based hand rub at points of patient evaluation?	70	5	20	5
4	Hospital provided verbal instructions, informational posters, cards, etc on IPC	75	0	25	0
5	Hospital has certified that HCWs are applying standard precautions	50	5	45	0
6	Isolation rooms for suspected/confirmed COVID-19 cases are well ventilated?	80	5	15	0
7	Hospital maintains a one-meter distance between beds regardless of COVID-19 status	50	10	30	10
8	Equipment used in the isolation unit is single-use or disposable or disinfected	60	10	25	5
9	Hospital routinely clean and disinfect surfaces	65	10	25	0
10	HCWs apply droplet and contact precautions before entering isolation room	80	5	15	0
11	HCWs are applying airborne precautions for aerosol-generating procedures.	75	10	15	0
12	Hospital has adequate PPE for use by HCWs	40	15	35	10
13	Hospital avoids moving/transporting patients unless medically indicated?	70	10	20	0
14	Hospital limits visitors and ensure visitors apply droplet and contact precautions	65	10	20	5
15	Hospital maintains a record of all persons entering the patient’s room	30	0	30	40
16	Hospital manages specimens, laundry, food, and waste according to IPC guidelines	60	20	20	0
17	Hospital has a team of HCWs to care exclusively for suspected/confirmed cases	80	5	10	5
18	Hospital ensured that staff receive training on various aspects of IPC	75	0	15	10
19	Hospital established a COVID-19 healthcare waste management protocol?	50	0	35	15
20	Isolation facility for COVID-19 has been secured with a perimeter fencing	55	5	15	25

NB: HCWs-healthcare workers, PPE-personal protective equipment, IPC-infection prevention and control, HCAI-healthcare associated infection.

### Case management

Regarding case management, 16 hospitals (80%) were providing patient care following national and international guidelines and had established triage protocol and implemented strategies for admission, internal transfer, referral, and discharge of COVID 19 patients (Table 5). However, only 45% of hospitals had established a triage station at the entrance to their facility and 55% of hospitals had made available staffed beds for admission of severe COVID-19 cases and designated an exclusive waiting and examination area for individuals presenting with respiratory symptoms and/or fever (Table 5).

**Table 5:** Implementation of COVID-19 case management readiness indicators among 20 hospitals in Nigeria.

Sn	Hospital readiness indicator	Completed	In progress	Due for review	Not started
		%	%	%	%
	**Case management**				
1	Hospital has implemented triage, early recognition, and source control (isolating patients with suspected COVID-19)	65	5	25	5
2	Hospital has established a triage station at the entrance of the health-care facility	45	15	30	10
3	Hospital has adopted screening questionnaires and post signs reminding symptomatic patients to alert HCWs	45	10	20	25
4	Hospital has designated an exclusive waiting and examination area for persons with respiratory symptoms and/or fever	55	0	20	25
5	Hospital has appointed a supervisor to oversee all triage operations	70	5	5	20
6	Hospital has established a triage protocol to identify acute respiratory infections	80	0	10	10
7	Hospital has implemented a strategy for the admission, internal transfer, referral, and discharge of COVID 19 patients	85	5	0	10
8	Hospital has made available staffed beds for admission of severe COVID-19 cases	55	10	20	15
9	Hospital has made available oxygen, respiratory support, and sedation for intubated patients	45	5	40	10
10	Hospital is providing care following national and international guidelines	80	0	20	0
11	Hospital has communicated admission criteria/triage logistics to relevant authorities	65	5	25	5
12	Hospital personnel are aware of protocols for off-license use of medicines.	25	10	30	35
13	Hospital has established a communication line (e.g., intercom, bed alarm) for patients to call the attention of staff on duty	30	15	20	35

### Human resources and staff welfare

About 70% of hospitals each had a clear policy on staff exposure and staff suspected or confirmed of COVID-19, 60% had identified number of HCW needed for operation of all units and 60% had prioritized staffing by unit and distributed personnel accordingly (Table 6). About 75% of hospitals had provided relevant training to ensure staff competency and safety. However, only 20% of hospitals had multidisciplinary psycho-social teams to support families of staff and patients, while just one (5%) hospital had addressed liability, insurance and temporary licensing issues regarding staff working outside their areas of expertise.

**Table 6:** Implementation of human resources and staff welfare readiness indicators among 20 hospitals in Nigeria.

Sn	Hospital readiness indicator	Completed	In progress	Due for review	Not started
		%	%	%	%
	**Human resource**				
1	Hospital has updated the staff contact list	35	5	35	25
2	Hospital anticipated staff absenteeism in advance and monitored it continuously	25	15	45	15
3	Hospital has a clear policy on staff exposure and staff suspected or confirmed of COVID-19	70	0	20	10
4	Hospital has identified number of HCWs needed for operation in all units	60	5	20	15
5	Hospital has prioritized staffing by unit and distributed personnel accordingly	60	5	30	5
6	Hospital has recruited and trained additional staff according to need	25	0	10	65
7	Hospital has familiarized staff to work in high-demand areas to support surge	45	5	30	20
8	Hospital has provided relevant training to ensure staff competency and safety	75	0	20	5
9	Hospital has multidisciplinary psycho-social teams to support families of staff and patients	20	0	25	55
10	Hospital addressed liability, insurance and temporary licensing issues regarding staff working outside their areas of expertise	5	0	20	75
11	Hospital has considered reassigning staff at high risk for complications of COVID-19	60	5	20	15
	**Staff welfare**				
1	Hospital has secure accommodation for staff involved in the care of COVID-19 cases	25	0	25	50
2	Hospital has plans to cater for feeding of staff dedicated for COVID-19 care	50	10	10	30
3	Hospital has plans for any prophylaxis for staff caring for COVID-19 patients	35	5	30	30
4	There is a special remuneration for staff involved in COVID-19 patient care	40	10	25	25
5	There is a health/life insurance for staff involved in COVID-19 patient care	10	10	30	50

Nine (50%) hospitals had plans to cater for feeding of staff dedicated to COVID-19 care (Table 6). Only 25% and 10% of hospitals had secured accommodation for staff involved in care of COVID-19 cases and provided health/life insurance for staff involved in COVID-19 patient care, respectively.

### Continuity of essential health services, and surveillance

Table 7 shows that most hospitals (70%) maintained essential hospital services while only 30% have listed all hospital services in order of priority. Regarding surveillance, 80% of hospitals ensured COVID-19 testing is done in line with national criteria but only eight hospitals (40%) each have appointed a hospital epidemiologist to coordinate surveillance and identified the information that needs to be collected and defined the objectives for its use (Table 7).

**Table 7:** Implementation of continuity of essential health services, and surveillance, early warning signs and monitoring readiness indicators among 20 hospitals in Nigeria.

Sn	Hospital readiness indicator	Completed	In progress	Due for review	Not started
		%	%	%	%
	**Continuity of essential health services**				
1	Hospital has listed all hospital services in priority order	30	5	45	20
2	Hospital provides services that facility must always provide	70	5	15	10
3	Hospital has identified the resources for continuity of essential hospital services	55	10	30	5
4	Hospital is familiar with preparedness for other contingencies (e.g., disasters/casualties)	45	15	35	5
	**Surveillance; early warning signs and monitoring**				
1	Hospital has appointed a hospital epidemiologist responsible for surveillance	40	20	20	20
2	Hospital has identified information needs to be collected and defined objectives for use	45	10	25	20
3	Hospital promotes reporting of unusual health events (COVID-19) by HCWs	70	10	20	0
4	Hospital has implemented data collection and reporting following national health directives?	50	10	35	5
5	Hospital complies with surveillance activities in line with national criteria	70	5	20	5
6	Hospital immediately investigate reports by HCWs of unusual health events	65	10	20	5
7	Hospital ensures prompt distribution of information on unusual health events and/or signals to decision makers	55	5	35	5
8	Hospital ensures COVID-19 testing is done in line with national criteria	80	5	5	10
9	Hospital has ensured all staff are conversant with standardized surveillance definitions, and unusual health events through training	65	10	20	5

NB: HCWs-healthcare workers

### Communication

Twelve hospitals (70.6%) ensured decisions related to management of COVID-19 are communicated to all relevant staff and stakeholders, 55% have briefed staff on their roles and responsibilities regarding care of COVID-19 and 60% have appointed a public information spokesperson to coordinate communication (Table 8).

**Table 8:** Implementation of communication readiness indicators among 20 hospitals in Nigeria.

Sn	Hospital readiness indicator	Completed	In progress	Due for review	Not started
		%	%	%	%
1	Hospital established mechanisms for sharing of information between the hospital administration, department, and facility staff	70	5	20	5
2	Hospital has briefed staff on their roles and responsibilities regarding care of COVID-19 patients	55	5	30	10
3	Decisions on clinical triage and related policies are communicated to relevant staff and stakeholders	65	10	20	5
4	Hospital has ensured analysis and reporting of information to supervisory stakeholders	55	0	35	10
5	Hospital has drafted key messages on COVID-19 targeting different audiences	50	5	35	10
6	Hospital has appointed a public information spokesperson to coordinate communication	60	10	20	10
7	Hospital has reliable communication systems and access to updated contact lists	35	10	35	20
8	Hospital has considered having a contact list with roles rather than specific people	20	10	35	35
9	Hospital is familiar with referral mechanisms from national level and related mechanisms	55	5	30	10
10	Hospital has mechanisms to streamline the sharing of information between the hospital administration, and departments/units/staffs	70	0	30	0

### Logistic, supplies and pharmaceuticals

About 40% of hospitals had an updated inventory of all equipment, supplies, and pharmaceuticals, and reordering mechanism. Only seven hospitals (35%) had estimated the consumption of essential equipment, supplies, and pharmaceuticals and 25% had established contingency agreements with vendors to ensure the procurement and prompt delivery of equipment, supplies, and other resources (Table 9).

**Table 9:** Implementation of logistic and management of supplies, including pharmaceuticals readiness indicators among 20 hospitals in Nigeria.

Sn	Hospital readiness indicator	Completed	In progress	Due for review	Not started
		%	%	%	%
1	There is an updated inventory of all equipment, supplies, and pharmaceuticals; and reordering mechanism	40	10	40	10
2	Hospital has estimated the consumption of equipment, supplies, and pharmaceuticals based on most likely outbreak scenario	35	10	40	15
3	Hospital has consulted with authorities to ensure the continuous provision of essential medications and supplies	40	15	35	10
4	Hospital has assessed the quality of contingency items prior to purchase	40	15	30	15
5	Hospital has contingency agreements with vendors to ensure the procurement and prompt supply of resources as required	25	5	40	30
6	Hospital has identified physical space within the hospital for the storage and stockpiling of additional supplies	65	10	25	0
7	Hospital has considered accessibility, security, temperature, ventilation, and humidity, and ensured an uninterrupted cold chain for essential refrigerated items	50	15	25	10
8	Hospital has stockpiled essential supplies and pharmaceuticals, with timely use of items to avoid loss due to expiration	40	15	35	10
9	The role of hospital pharmacy in providing pharmaceuticals for home or other alternative treatment is defined	50	10	25	15
10	There is a mechanism for timely maintenance/repair of the essential equipment and deferment of non-essential maintenance	45	20	30	5
11	Hospital has established a contingency transportation strategy for continual patient transfers, such as designated ambulance teams	45	25	15	15
12	Hospital has ensured there is a policy in place for managing donations of medical supplies, food for staff, etc.	45	15	20	20

### Laboratory services and essential support services

Sixteen (80%) hospitals had ensured the continuous availability of basic laboratory testing, but only seven hospitals (40%%) had established a laboratory referral pathway for the identification, confirmation, and monitoring of COVID-19 (Table 10). About 70% of hospitals had trained staff on specimen handling, packaging, and transportation procedures for specimen referrals, 50% of hospitals had established a biosafety level 2 or higher laboratory for diagnosis of infectious diseases, and 40% had the capability to test for COVID-19.

**Table 10:** Implementation of laboratory services and essential support services readiness indicators among 20 hospitals in Nigeria.

Sn	Hospital readiness indicator	Completed	In progress	Due for review	Not started
		%	%	%	%
	**Laboratory services**				
1	Hospital has ensured the continuous availability of basic laboratory testing	80	0	10	10
2	Hospital has identified essential laboratory supplies and resources and ensure their continuous availability	70	5	15	10
3	Hospital has identified back-up laboratory personnel and/or alternative laboratory services	55	5	15	25
4	Hospital has mechanisms for the prompt provision of laboratory data to the physicians and health authorities	50	5	35	10
5	Hospital has prioritized testing for respiratory viruses (e.g., COVID-19)	55	15	20	10
6	Hospital has a referral pathway for the identification, confirmation, and monitoring of COVID-19	40	5	25	30
7	Hospital has trained staff on packaging and transportation procedures for specimen referrals	70	5	20	5
8	Hospital has established a biosafety level 2 or above laboratory for diagnosis of infectious diseases	50	0	40	10
9	Hospital has the capability to test for COVID-19	40	0	15	45
10	There is a focal person in charge of running samples from COVID-19 patients for other routine investigations	65	0	5	30
	**Essential support services**				
1	Hospital has estimated the additional supplies required by the support services and has a mechanism to ensure the continuous availability of these supplies	40	5	40	15
2	Hospital has enabled the adaptation of the support services to cope with increased demand	50	5	35	10
3	Hospital has anticipated the impact of COVID-19 on hospital food supplies; and taken measures to ensure availability of food	40	15	30	15
4	Hospital has ensured the availability of appropriate back-up arrangements for water, power, and oxygen	50	10	35	5
5	Hospital has solicited the input of hospital security in identifying potential security constraints and optimizing the control of facility access, and resources	65	20	10	5
6	Hospital has designated an area for use as a temporary morgue; ensure the adequate supply of body bags and shroud packs	40	5	15	40
7	Hospital has formulated a post-mortem care contingency plan with appropriate partners (e.g., undertakers, funeral services)	30	5	15	50

About 50% of hospitals had ensured the availability of appropriate back-up arrangements for water, power, and oxygen. Less than 40% of hospitals had designated an area for use as a temporary morgue and formulated a post-mortem care contingency plan with appropriate partners.

### Availability of critical items

Most of the hospitals evaluated reported inadequate supplies of critical items related to COVID-19 care. Only three hospitals reported having adequate supplies of PPE. Less than 25% of hospitals had adequate supplies of fingertip pulse oximeters and nasal prongs for oxygen delivery (Table 11).

**Table 11:** Availability of critical items for COVID-19 response among 20 hospitals in Nigeria.

	Hospital readiness indicator	Yes, adequate	Yes, but not adequate	No
		%	%	%
1	Hospital has supplies of full PPEs	15	60	25
2	Hospital has supplies of surgical mask	10	65	25
3	Hospital has supplies of N95 mask (particulate respirator)	20	55	25
4	Hospital has fingertip pulse oximeter for management of COVID-19 patients	15	65	20
5	Hospital has oxygen concentrators for management of COVID-19 patients	30	40	30
6	Hospital has ventilators for management of adults and paediatric age group COVID-19 patients	30	50	20
7	Hospital has adequate quantity of CPAP 10 machine, with twin flowmeters for management of COVID-19 patients	50	25	25
8	Hospital has CPAP unit with nasal tubing and mask for adult for management of COVID-19 patients	45	30	25
9	Hospital has Flowmeter, Thorpe tube, for oxygen 0-15L/min for management of COVID-19 patients	35	45	20
10	Hospital has suction pump, mechanical (twin pump) for management of COVID-19 patients	30	45	25
11	Hospital has high flow nasal cannula for management of COVID-19 patients	25	30	45

NB: PPE-personal protective equipment, CPAP-continuous positive airway pressure

## Discussion

The WHO COVID-19 hospital readiness checklist was developed for hospital leaders to rapidly determine their current capacities and gaps in services necessary to respond to the COVID-19 pandemic, to help them identify major areas that require investment and action, and to develop plans to improve hospital readiness [11].

Our study findings have revealed gaps in infrastructure, equipment, human resources, processes, and procedures related to COVID-19 response among both secondary and tertiary health facilities in Nigeria. These gaps persisted even among hospitals evaluated five to eight months after the first case of COVID-19 was reported in the country.

Any successful response to a public health threat is driven by an evidence-based emergency response plan (ERP) implemented by an incident management team and coordinated through an emergency operations centre (EOC) [12, 13]. Even though majority of the hospitals surveyed had a COVID-19 incident management teams at the time of evaluation, less than eight hospitals had a COVID-19 ERP or EOC. Lack of planning and inadequate coordination of information and resources for the COVID-19 response as provided by an ERP and EOC could partly explain the predominant ‘moderately ready’ and ‘not ready’ COVID-19 readiness scores by hospitals in our study. Pre-existing gaps in various service delivery elements also played a major role in the poor readiness scores. For instance, for each hospital surveyed, there was a median of 15 isolation beds, four ICU beds, four ventilators and between one to seven COVID-19-related specialist human resources. This suggest that most hospitals even lacked resources for routine patient care before the COVID-19 pandemic and were not prepared to accommodate a surge in cases of COVID-19.

In the face of the COVID-19 pandemic, an effective hospital emergency response should sustain all essential health services, be able to expand hospital facilities and resources for surge of severe and critical cases of COVID-19, and provide a safe and motivating environment for staff [5]. Although over 80% of hospitals in our study had cancelled non-essential health services, while about 70% had maintained essential health services, majority of hospitals had no plans to expand hospital in-patient capacity, no plans for critical care of COVID-19 patients and no plans for a temporary morgue for deceased patients. There were significant gaps in logistics and supplies as over 60% of hospitals had neither estimated consumption of hospital commodities nor established contingency agreement for procurements. Staff welfare was also inadequate as only five of the 20 hospitals surveyed had secured accommodation for staff involved in COVID-19 care, only four hospitals had multidisciplinary psycho-social teams to support families of staff and patients, and only two hospitals had provided health or life insurance for staff involved in COVID-19 patient care. Majority of hospitals surveyed reported a lack or paucity of critical items for COVID-19 response. For instance, about 65% of hospitals reported not having adequate supplies of full PPE and surgical masks for use by HCWs, while 25% of hospitals did not have supplies of PPE.

Our study findings also reveal that COVID-19 instigated some improvement in hospital infrastructure, especially among hospitals who established isolation units and procured ventilators for the first time due to the COVID-19 pandemic. The highest readiness scores were reported for IPC and surveillance. The reasons for these high scores are not obvious from our study findings. Possibly, hospitals prioritized implementation of activities under these components as they are related to detection and prevention of COVID-19 infection among patients and staff. However, reported implementation does not necessarily imply compliance. For instance, only 50% of hospitals surveyed had certified that their HCWs were applying standard precautions. The reported inadequacies in PPEs also suggest that compliance with IPC was limited among HCWs.

Our study findings are comparable to other published literature from within and outside Nigeria. At the onset of the COVID-19 pandemic, the WHO assessed the readiness status of 48 African countries to respond to COVID-19, revealing that 8 (16.7%) had adequate readiness, 33 (68.8%) had moderate readiness and 7 (14.6%) had limited readiness status [10]. Nigeria was ‘moderately’ ready, but the country scored below average in four out of the nine pandemic readiness checklists, including surveillance, case management, IPC, and rapid response team. A mid-term (February to June 2020) review of Nigeria’s COVID-19 multisectoral response identified challenges relating to delay in supply of PPEs, inadequate health infrastructure, disrupted supply chains and increased cost of procurement, among other challenges [14]. The challenges of inadequate PPEs, unpaid COVID-19 hazard allowances and other poor welfare issues, compelled resident doctors in Nigeria to embark on a national strike in June 2020 [15].

Other national studies from developing countries, including Malawi [16], Kenya [17], India [18], and Nepal [19], have largely reported variable levels of unpreparedness for COVID-19 response due to gaps in availability of PPEs, inadequate isolation facilities and poor readiness for critical care of COVID-19 patients, among other challenges. However, COVID-19 also overwhelmed strong health systems of developed countries. A national review of United States of America (USA) hospital experiences to the COVID-19 pandemic conducted in March 2020, revealed that majority of US hospitals had challenges related to patient care, expanding facility capacity, availability of PPEs and staff safety [20]. A COVID-19 preparedness health facility assessment from Ukraine revealed that less than 50% of hospitals had isolation units and over 80% of hospitals lacked surgical masks for use by HCWs [21]. Another study from Iran reported an overall readiness score of 42% among 24 hospitals, with gaps in almost every element of the readiness checklist [22]. In a survey on continuity of essential health services during COVID-19 conducted among 105 countries between May and July 2020, disruptions in services were more common among low- and middle-income than in high-income countries [23]. Among the 105 countries surveyed, 90% reported at least partial disruptions in one or more essential health services, 44% reported insufficient PPEs for HCWs and 30% reported interruptions in the supply of medical equipment and health products.

Our study has some limitations. The date of assessment of hospital readiness was not uniform among hospitals surveyed, making it impossible to assess true variability of readiness among hospitals. However, the variability in the date of evaluation provides a picture of how hospitals have responded within two to eight months after the first case of COVID-19 was reported in Nigeria. The checklist was completed by designated representatives of the hospital and their responses were not independently verified. Consequently, the possibilities of response bias cannot be excluded. We only included three secondary health facilities in our study, and our findings among these hospitals may not be generalizable to similar healthcare facilities in the country.

Notwithstanding these limitations, our study is the first from Nigeria evaluating readiness of hospitals to respond to the COVID-19 pandemic. Our results reveal that most hospitals surveyed were not adequately ready for the COVID-19 pandemic, with variability among hospitals and a general poor state of readiness in relation to surge capacity, critical care, human resources, staff welfare and availability of critical items for COVID-19 response. While Nigeria continues to improve its hospital readiness for COVID-19, [14] sustainable interventions that address challenges related to management of severe and critical cases in health care facilities, including improved health workforce for epidemic response, construction of well-equipped ICUs in all states of the federation, provision of adequate supplies of PPEs for HCWs and attention to improvement of staff welfare and safety are required.

## Supporting information

S1 Text Hospital readiness survey questionnaire during COVID-19 in Nigeria. (DOCX)

S1 File Hospital readiness survey data. (SAV)
