# Supplementary material for: A national survey of hospital readiness during the COVID-19 pandemic in Nigeria
Source: PLoS One. 2021 Sep 21;16(9):e0257567. doi: 10.1371/journal.pone.0257567 (PMC8454967; doi:10.1371/journal.pone.0257567)
Supplement: S1 Text — (DOCX) [file pone.0257567.s001.docx]

**Hospital readiness checklist**

**Research Topic**: A rapid assessment of preparedness of hospitals in Nigeria to respond to the COVID-19 pandemic.

**DESCRIPTION OF HOSPITAL**

1. Evaluation Date………………………………………………….
2. Name of Hospital…………………………………………………………………
3. Administrative Status (Tick One): a. State [] b. Private [] c. Federal [] d. Other []
4. Number of Beds……………………
5. Does your facility have a functional intensive care unit (ICU)? a. Yes [] b. No []
6. If yes, when was the ICU established (specify month/year) …………………………
7. Intensive Care Unit (ICU) Beds……Adult ICU Bed…… Paediatric ICU Beds………
8. Do you have a ventilator in your hospital a. Yes [] b. No []
9. If yes,
   1. Number of functional ventilators in your hospital………………….?
   2. When was (were) the ventilator(s) purchased?
      1. How many before COVID-19 pandemic []……………….
      2. ii. How many during COVID-19 pandemic []……………..
10. Do You Have Isolation Ward/Unit in Your Hospital a. Yes [] b. No []?
11. If Yes,
    1. Specify month and year it was established …………?
    2. How many beds are in the isolation unit…………………?
    3. How many adult beds…………? How many Paediatric beds……..?
    4. How many beds in single rooms? ………………………………
12. Has your facility seen any suspected case of COVID-19 since the pandemic started?
    1. Yes [] b. No []
13. Do you currently have a suspected or confirmed case (or cases) of COVID-19 isolated in your hospital?
    1. Yes (specify number suspected…; specify number confirmed…..)
    2. No
14. Does your hospital have a functional infection prevention and control (IPC) committee?
    1. Yes, functional b. Yes, not functional c. No IPC committee
15. Do you have any of the following health professionals as employees in your hospital?

| Sn | Profession | Yes | No | If, yes how many (indicate full and part-time staff separately)? | How many of each group have been trained on COVID-19? |
| --- | --- | --- | --- | --- | --- |
| 1 | Anaesthesiologist |  |  |  |  |
| 2 | Infectious disease specialists |  |  |  |  |
| 3 | Public health physician/  hospital epidemiologists |  |  |  |  |
| 4 | Pulmonologists |  |  |  |  |
| 5 | Clinical microbiologists |  |  |  |  |
| 6 | Infection control officer/nurse |  |  |  |  |

1. Designation of evaluators
   1. …………………………………. b. …………………………………

c. ……………………………………

**INCIDENT MANAGEMENT TEAM**

A well-functioning hospital incident management system is essential for the effective management of emergency operations.

| Sn. | Indicator | Not started | Due for review | In progress | Completed |
| --- | --- | --- | --- | --- | --- |
| 1 | Do you have a hospital emergency Response Plan? |  |  |  |  |
| 2 | Do you have a hospital emergency operation centre? |  |  |  |  |
| 3 | Has your hospital established an incident management committee or response team for COVID-19? |  |  |  |  |
| 4 | If yes, who is the lead of your incident management team or the incident manager (specify designation and profession)? |  | | | |

**SURGE CAPACITY**

Surge capacity is the ability of a health service to expand beyond its normal capacity to meet an increased demand for clinical care. COVID-19 cases may cause rapid increase in demand over a prolonged period of time (“rising tide” as opposed to “big bang” of a sudden-onset disaster)

| Sn. | Indicator | Not started | Due for review | In progress | Completed |
| --- | --- | --- | --- | --- | --- |
| 1 | Has your hospital estimated its capacity to accommodate a surge in cases of COVID-19? |  |  |  |  |
| 2 | Has your hospital identified ways of expanding hospital in-patient capacity (including physical space, staff, supplies and processes)? |  |  |  |  |
| 3. | Has your hospital identified potential gaps in the provision of health care, with an emphasis on critical care? |  |  |  |  |
| 4 | Has your hospital developed strategies to address challenges associated with critical care? |  |  |  |  |
| 5 | Has your hospital considered releasing additional capacity by outsourcing care of non-critical patients to appropriate alternative treatment sites? |  |  |  |  |
| 6 | Has your hospital, in conjunction with local authorities, identified additional sites for conversion to patient care units (e.g. convalescent homes, hotels, schools, community centres, gymnasiums)? |  |  |  |  |
| 7 | Has your hospital considered cancelling nonessential services (e.g. elective surgery) when necessary? |  |  |  |  |
| 8 | Has your hospital adapted admission and discharge criteria and prioritize patients and clinical interventions according to available treatment capacity and demand? |  |  |  |  |
| 9 | Has your hospital considered the possibility of setting up isolation tents with beds to cater for a surge in patients’ numbers? |  |  |  |  |

**INFECTION PREVENTION AND CONTROL**

An operational infection prevention and control (IPC) programme is essential to minimize the risk of transmission of healthcare-associated infection to patients, hospital staff, and visitors.

| Sn. | Indicator | Not started | Due for review | In progress | Completed |
| --- | --- | --- | --- | --- | --- |
| 1 | Has your hospital sensitized health care workers (HCW), patients, and visitors about respiratory and hand hygiene and prevention of healthcare-associated infections? |  |  |  |  |
| 2 | Does you hospital provide soap and water for hand hygiene? |  |  |  |  |
| 3 | Does your hospital provide alcohol-based rub at points of patient evaluation? |  |  |  |  |
| 4 | Has your hospital provided verbal instructions, informational posters, cards, etc on IPC? |  |  |  |  |
| 5 | Has your hospital certified that HCW are applying standard precautions for all patients? |  |  |  |  |
| 6 | Are isolation rooms for suspected or confirmed COVID-19 cases well ventilated? |  |  |  |  |
| 7 | Does your hospital ensure a one-meter distance between beds regardless of whether patients are suspected of having COVID-19? |  |  |  |  |
| 8 | Does your hospital ensure equipment used in the isolation unit is either single-use and disposable OR if equipment (e.g., stethoscopes, blood pressure cuffs, thermometers, food trays) needs to be shared among patients, they are cleaned and disinfected between use for each patient? |  |  |  |  |
| 9 | Does your hospital routinely clean and disinfect surfaces with which the patient is in contact.? |  |  |  |  |
| 10 | Does your hospital ensure that HCWs are applying droplet and contact precautions before entering the room where suspected or confirmed infectious diseases patients (including COVID-19 patients) are admitted? |  |  |  |  |
| 11 | Does your hospital ensure that HCWs are applying airborne precautions for aerosol-generating procedures, such as tracheal intubation, non-invasive ventilation, tracheotomy, cardiopulmonary resuscitation, etc? |  |  |  |  |
| 12 | Does your hospital have adequate personal protective equipment (PPE) for use by healthcare workers? |  |  |  |  |
| 13 | Does your hospital avoid moving and transporting patients out of their room or area unless medically indicated? |  |  |  |  |
| 14 | Does your hospital limit visitors to those essential for patient support and ensure visitors apply droplet and contact precautions? |  |  |  |  |
| 15 | Does your hospital maintain a record of all persons entering the patient’s room, including all staff and visitors? |  |  |  |  |
| 16 | Does your hospital manage laboratory specimens, laundry, food service utensils, and medical waste following safe routine procedures according to IPC guidelines? |  |  |  |  |
| 17 | Does your hospital have a team of HCWs designated to care exclusively for suspected or confirmed cases to reduce the risk of transmission? |  |  |  |  |
| 18 | Has your hospital ensured that staff (HCW, cleaning personnel) receive training on standard, contact, droplets, and airborne precautions (including correct use of PPE, donning and doffing, Masks tested for fitting, hand hygiene, respiratory hygiene, etc.)? |  |  |  |  |
| 19 | Has your hospital established a COVID-19 healthcare waste management protocol? |  |  |  |  |
| 20 | Has the isolation facility for COVID-19 been secured with a perimeter fencing? |  |  |  |  |

**CASE MANAGEMENT**

An efficient and accurate triage system and an organized in-patient management strategy are required to ensure adequate treatment of COVID-19 acute respiratory infection.

| SN | indicator | Not started | Due for review | In progress | completed |
| --- | --- | --- | --- | --- | --- |
| 1 | Has your hospital instituted mechanisms to implement triage, early recognition, and source control (isolating patients with suspected COVID-19)? |  |  |  |  |
| 2 | Has your hospital established a well-equipped triage station at the entrance of the health-care facility, supported by trained staff? |  |  |  |  |
| 3 | Has your hospital instituted the use of screening questionnaires according to the updated case definition and post signs in public areas reminding symptomatic patients to alert HCWs? |  |  |  |  |
| 4 | Has your hospital designated an exclusive waiting and examination area for individuals presenting with respiratory symptoms and/or fever? |  |  |  |  |
| 5 | Has your hospital appointed a triage supervisor responsible for overseeing all triage operations? |  |  |  |  |
| 6 | Has your hospital established a triage protocol aimed at ensuring that cases of acute respiratory infection are recognized? |  |  |  |  |
| 7 | In coordination with local health authorities, has your hospital implemented a strategy for the admission, internal transfer, referral, and discharge of COVID 19 patients? |  |  |  |  |
| 8 | Has your hospital certified the availability of staffed beds for the admission of severe COVID-19 acute respiratory infection cases requiring supportive care and continuous/regular monitoring? |  |  |  |  |
| 9 | Has your hospital ensured the availability of oxygen and means of respiratory support, as well as sufficient sedation for intubated patients? |  |  |  |  |
| 10 | Is your hospital providing patient care following national and international guidelines? |  |  |  |  |
| 11 | Has your hospital communicated admission criteria and triage logistics (e.g., location, routes of entry/exit) to the relevant hospital personnel, referring hospitals and clinics, pre-hospital networks, and ambulance services? |  |  |  |  |
| 12 | Has your hospital ensured that healthcare personnel are aware of protocols for off-license use of medicines, which should be done against observational trial protocol and outcomes recorded against standardized variables? |  |  |  |  |
| 13 | Has your hospital planned to establish a communication line (e.g. intercom, dedicated mobile number, bed alarm) for patients to call the attention of staff on duty? |  |  |  |  |

**HUMAN RESOURCES**

Adapted human resource management is required to ensure adequate staff capacity and continuity of operations in response to an increased demand for human resources, while maintaining the identified essential services

| SN. | Indicator | Not started | Due for review | In progress | Completed |
| --- | --- | --- | --- | --- | --- |
| 1. | Has your hospital updated the staff contact list? |  |  |  |  |
| 2 | Has your hospital anticipated staff absenteeism (or staff leave) in advance and monitored it continuously? |  |  |  |  |
| 3 | Has your hospital established a clear policy (the policy should define levels of exposure) to monitor and manage staff suspected or confirmed of having COVID-19 or who have had exposure to a confirmed, probable or suspected COVID-19 patient? |  |  |  |  |
| 4 | For each unit or service, has your hospital identified the minimum number of health-care workers and other hospital staff needed to ensure the sufficient operation of the unit or service? |  |  |  |  |
| 5 | Has your hospital prioritized staffing needs by unit or service and distribute personnel accordingly? |  |  |  |  |
| 6 | Has your hospital recruited and trained additional staff (e.g. retired staff, reserve military personnel, university affiliates/students, community volunteers) according to the anticipated need? |  |  |  |  |
| 7 | Has your hospital, familiarized ward staff to work in high-demand areas (e.g. infectious disease wards, emergency and intensive care units) to support surge? |  |  |  |  |
| 8 | Has your hospital provided training and exercises relevant to areas of need, including infection prevention and control, clinical management, to ensure staff competency and safety? |  |  |  |  |
| 9 | Has your hospital ensured the availability of the services of multidisciplinary psycho-social support teams for the families of staff and patients, including social workers, counsellors, interpreters and clergymen? |  |  |  |  |
| 10 | Has your hospital addressed liability, insurance and temporary licensing issues with respect to staff who may be working outside their areas of expertise? |  |  |  |  |
| 11 | Has your hospital considered reassigning staff at high risk for complications of COVID-19 acute respiratory infection? |  |  |  |  |

**CONTINUITY OF ESSENTIAL HEALTH SERVICES AND PATIENT CARE**

An outbreak of COVID-19 will not dispel an already existing need for essential medical and surgical care (e.g. emergency services, urgent surgical operations, maternal and child-care); hence, it is necessary to ensure the continuity of essential health services.

| SN. | Indicator | Not started | Due for review | In progress | Completed |
| --- | --- | --- | --- | --- | --- |
| 1 | Has your hospital listed all hospital services in priority order? |  |  |  |  |
| 2 | Has your hospital identified and maintained the hospital services that your facility must provide at all times and under any circumstances? |  |  |  |  |
| 3 | Has your hospital identified the resources (human resources and logistics) needed to ensure the continuity of the identified essential hospital services? |  |  |  |  |
| 4 | Is your hospital familiar with preparedness mechanisms across the local health-care network for other high- demand contingencies (e.g. disasters or mass- casualty incidents)? |  |  |  |  |

**SURVEILLANCE, EARLY WARNING SIGN AND MONITORING**

Health-care workers recognizing and immediately reporting unusual health events (e.g., clusters of cases, atypical clinical presentations, etc.) occurring in health-care facilities are the cornerstone of the early warning function. In addition to serving the early warning function, the laboratory and epidemiological data obtained through systematic collection and analysis allows the public health authorities to monitor.

| SN. | Indicator | Not started | Due for review | In progress | Completed |
| --- | --- | --- | --- | --- | --- |
| 1 | Has your hospital appointed a hospital epidemiologist with the overall responsibility for activities related to early warning and surveillance in the hospital? |  |  |  |  |
| 2 | Has your hospital identified the information that needs to be collected and define the objectives for its use? |  |  |  |  |
| 3 | Does your hospital promote the reporting of unusual health events (COVID-19) by health-care workers by establishing communication channels and procedures within the hospital and with public health authorities? |  |  |  |  |
| 4 | Has your hospital implemented data collection and reporting mechanisms following the national health policy and directives? |  |  |  |  |
| 5 | Does your hospital comply with standardized case definitions, recommended levels of surveillance, and triggers for surveillance escalation or de-escalation in accordance with national criteria? |  |  |  |  |
| 6 | Does your hospital immediately investigate reports by health care workers of unusual health events and/or unusual signals detected through monitoring activities? |  |  |  |  |
| 7 | Has your hospital ensured prompt distribution to hospital clinicians, front-line workers, and other relevant decision- makers of information obtained through monitoring activities and/or the investigation of unusual health events and/or signals? |  |  |  |  |
| 8 | Has your hospital ensured that testing of persons hospitalized for COVID-19 complies with the standardized case definitions, recommended levels of surveillance, and triggers for surveillance escalation or de- escalation in accordance with the national criteria? |  |  |  |  |
| 9 | Has your hospital ensured all staff are conversant with standardized case definitions, recommended levels of surveillance and triggers for surveillance escalation or de-escalation, in accordance with the national criteria, as well as recognizing unusual health events through training? |  |  |  |  |

**COMMUNICATION**

Accurate and timely communication is necessary to ensure informed decision-making, effective collaboration and cooperation, and public awareness and trust.

| SN. | Indicator | Not started | Due for review | In progress | Completed |
| --- | --- | --- | --- | --- | --- |
| 1 | Has your hospital established mechanisms of communication to streamline the sharing of information between the hospital administration, department/unit heads, and facility staff? |  |  |  |  |
| 2 | Has your hospital briefed the hospital staff on their roles and responsibilities in the management of COVID-19 under the incident management system? |  |  |  |  |
| 3 | Has your hospital ensured that all decisions on clinical triage, patient prioritization (e.g., adapted admission and discharge criteria), infection prevention and control measures, and policies related to case management and hospital epidemiology are communicated to all relevant staff and stakeholders? |  |  |  |  |
| 4 | Has your hospital ensured the collection, processing, and reporting of information to supervisory stakeholders (e.g., public health authorities), and through them to neighbouring hospitals, private practitioners, and pre-hospital networks? |  |  |  |  |
| 5 | Has your hospital drafted in advance, key messages, addressing a variety of COVID-19-related scenarios with different target audiences in mind (e.g., patients, visitors, staff, the general public, media)? |  |  |  |  |
| 6 | Has your hospital appointed a public information spokesperson to coordinate communication with the public, the media, and health authorities? |  |  |  |  |
| 7 | Has your hospital ensured reliable and sustainable primary and back- up communication systems (e.g., landlines, the internet, mobile devices, pagers, satellite telephones, two-way radio equipment, unlisted numbers) and access to updated contact lists? |  |  |  |  |
| 8 | Has your hospital considered having a contact list with roles rather than specific people? |  |  |  |  |
| 9 | Is your hospital familiar with referral mechanisms established at the national level and related communication mechanisms? |  |  |  |  |
| 10 | Has your hospital established mechanisms of communication to streamline the sharing of information between the hospital administration, department/unit heads, and facility staff? |  |  |  |  |

**LOGISTIC AND MANAGEMENT OF SUPPLIES, INCLUDING PHARMACEUTICALS**

The continuity of hospital services and the availability of essential equipment and supplies, including pharmaceuticals, require a proactive approach to resource and facility management.

| SN. | Indicator | Not started | Due for review | In progress | Completed |
| --- | --- | --- | --- | --- | --- |
| 1 | Has your hospital developed/maintained an updated inventory of all equipment, supplies, and pharmaceuticals; established a shortage alert and reordering mechanism? |  |  |  |  |
| 2 | Has your hospital estimated the consumption of essential equipment, supplies, and pharmaceuticals (e.g., amount used per week) based on most likely outbreak scenario? |  |  |  |  |
| 3 | Has your hospital consulted with authorities to ensure the continuous provision of essential medications and supplies (e.g. institutional and central stockpiles, emergency agreements with local suppliers, donations)? |  |  |  |  |
| 4 | Has your hospital assessed the quality of contingency items prior to purchase; request quality certification? |  |  |  |  |
| 5 | Has your hospital established contingency agreements (e.g., memorandum of understanding, mutual aid agreement) with vendors to ensure the procurement and prompt delivery of equipment, supplies, and other resources in times of shortage? |  |  |  |  |
| 6 | Has your hospital identified physical space within the hospital for the storage and stockpiling of additional supplies? |  |  |  |  |
| 7 | Has your hospital considered the following factors: accessibility, security, ambient temperature, ventilation, light exposure, and humidity. Ensured an uninterrupted cold chain for essential items requiring refrigeration.? |  |  |  |  |
| 8 | Has your hospital stockpiled essential supplies and pharmaceuticals according to recommended guidelines and ensured the timely use of stockpiled items to avoid loss due to expiration? |  |  |  |  |
| 9 | Has your hospital defined the role of the hospital pharmacy in providing pharmaceuticals for cases treated at home or other alternative treatment sites? |  |  |  |  |
| 10 | Has your hospital ensured a mechanism for the prompt maintenance and repair of the equipment required for essential services and postponed non-essential maintenance and repairs? |  |  |  |  |
| 11 | Has your hospital coordinated with pre-hospital networks and transportation services in establishing a contingency transportation strategy to ensure continual patient transfers, such as designated ambulance teams (as the outbreak grows, the strategy may need to change)? |  |  |  |  |
| 12 | Has your hospital ensured there is a policy in place for managing donations of medical supplies, food for staff, etc.? |  |  |  |  |

**LABORATORY SERVICES**

Maintenance of the essential laboratory services is necessary for the appropriate clinical management of both pandemic and other patients, as well as for the hospital- based surveillance of COVID-19

| SN. | Indicator | Not started | Due for review | In progress | Completed |
| --- | --- | --- | --- | --- | --- |
| 1 | Has your hospital ensured the continuous availability of basic laboratory testing (e.g., complete blood count, biochemistry profile, electrolytes, blood gas analysis, blood culture, and sputum examination)? |  |  |  |  |
| 2 | Has your hospital identified essential laboratory supplies and resources and ensure their continuous availability? |  |  |  |  |
| 3 | Has your hospital identified back-up laboratory personnel and/or alternative laboratory services? |  |  |  |  |
| 4 | For hospital-based surveillance, has your hospital ensured mechanisms for the prompt provision of laboratory data to the physicians and health authorities responsible for clinical management and surveillance? |  |  |  |  |
| 5 | Has your hospital prioritized testing for respiratory viruses (e.g., COVID-19) according to clinical requirements and hospital-based surveillance needs? |  |  |  |  |
| 6 | Has your hospital established a laboratory referral pathway for the identification, confirmation, and monitoring of COVID-19, (including changes in virus characteristics, such as virulence, transmissibility, and antivirus resistance)? |  |  |  |  |
| 7 | Has your hospital established and trained staff on packaging and transportation procedures for specimen referrals in accordance with national and international transport regulations and requirements? |  |  |  |  |
| 8 | Has your hospital established a biosafety level 2 or above laboratory for diagnosis of infectious diseases? |  |  |  |  |
| 9 | Does your hospital have the capability to test for COVID-19? |  |  |  |  |
| 10 | Is there a focal person in charge of running samples from COVID-19 patients for other routine investigations? |  |  |  |  |

**ESSENTIAL SUPPORT SERVICES**

To optimize patient care during the COVID-19 outbreak, it is necessary to identify and maintain essential support services, such as those for laundry, cleaning, waste management, dietary services, and security.

| SN. | Indicator | Not started | Due for review | In progress | Completed |
| --- | --- | --- | --- | --- | --- |
| 1 | Has your hospital estimated the additional supplies required by the support services and introduced a mechanism to ensure the continuous availability of these supplies? |  |  |  |  |
| 2 | Has your hospital enabled the adaptation of the support services to cope with increased demand? |  |  |  |  |
| 3 | Has your hospital anticipated the impact of COVID-19 on hospital food supplies; and taken proactive measures to ensure the availability of food? |  |  |  |  |
| 4 | Has your hospital ensured the availability of appropriate back-up arrangements for essential lifelines, including water, power, and oxygen? |  |  |  |  |
| 5 | Has your hospital solicited the input of hospital security in identifying potential security constraints and optimizing the control of facility access, essential pharmaceutical stocks, patient flow, traffic, and parking? |  |  |  |  |
| 6 | Has your hospital designated an area for use as a temporary morgue; ensure the adequate supply of body bags and shroud packs? |  |  |  |  |
| 7 | Has your hospital formulated a post-mortem care contingency plan with appropriate partners (e.g., undertakers, funeral services)? |  |  |  |  |

**STAFF WELFARE**

To ensure safety and welfare of staff who care for COVID-19 patients, it is necessary to provide incentives, compensations and other measures that serve to motivate staff and prevent transmission of the virus.

| SN. | Indicator | Not started | Due for review | In progress | Completed |
| --- | --- | --- | --- | --- | --- |
| 1 | Has your hospital made provision for secure accommodation for staff involved in management of COVID-19 patients? |  |  |  |  |
| 2 | Has your hospital made plans to cater for feeding of staff dedicated for COVID-19 management? |  |  |  |  |
| 3 | Has your hospital made plans for any prophylaxis as the case may be for staff catering for COVID-19 patients? |  |  |  |  |
| 4 | Is there any provision for special remuneration for staff involved in COVID-19 patient care? |  |  |  |  |
| 5 | Is there any provision of health/life insurance for staff involved in COVID-19 patient management? |  |  |  |  |

**CRITICAL ITEMS**

Procurement and availability of critical items such as consumables and equipment for isolation and management of suspected or confirmed cases of COVID-19 is an essential ingredient of hospital’s readiness to respond to COVID-19.

| Sn | Indicator | Yes, adequate | Yes, but not adequate | No |
| --- | --- | --- | --- | --- |
| 1 | Does your hospital have supplies of full PPEs (including gowns, head gear, gloves, shoe cover, googles (or face shield) and face mask)? |  |  |  |
| 2 | **Does your hospital have supplies of surgical mask?** |  |  |  |
| 3 | **Does your hospital have supplies of N95 mask? (particulate respirator)?** |  |  |  |
| 4 | **Does your hospital have fingertip p**ulse oximeter for management of COVID-19 patients? |  |  |  |
| 5 | **Does your hospital have oxygen concentrators for management of COVID-19 patients?** |  |  |  |
| 6 | **Does your hospital have ventilators for adults and paediatric age group for management of COVID-19 patients?** |  |  |  |
| 7 | **Does your hospital have adequate quantity of** **CPAP 10 machine, w/twin flowmeters?** |  |  |  |
| 8 | **Does your hospital have CPAP unit w/nasal tubing and mask for adult for management of COVID-19 patients??** |  |  |  |
| 9 | **Does your hospital have Flowmeter, Thorpe tube, for oxygen 0-15L/min? for management of COVID-19 patients?** |  |  |  |
| 10 | **Does your hospital have suction pomp, mechanical (twin pump) for management of COVID-19 patients?** |  |  |  |
| 11 | **Does your hospital have high flow nasal cannula for management of COVID-19 patients?** |  |  |  |
